# Blurry Diagnosis of Ocular Syphilis

**DOI:** 10.7759/cureus.29950

**Published:** 2022-10-05

**Authors:** Roudabeh Kiani, Abolfazl Ahmady, Kunjal Luhadia, Mohamed Abugrin, Jaswinder Virk, Kanica Yashi

**Affiliations:** 1 Internal Medicine, Bassett Medical Center, Cooperstown, USA; 2 Cardiology, State University of New York (SUNY) Upstate Medical University, Syracuse, USA

**Keywords:** penicillin, rash, msm, ocular, syphilis

## Abstract

The incidence of syphilis is on the rise worldwide which may be due to increased diagnosis. Given the wide variety of presenting symptoms, syphilis can pose many diagnostic and therapeutic difficulties for doctors. In turn, these difficulties frequently lead to a noticeable delay in patient care. Here we present a case of ocular syphilis in a homosexual male patient presenting with uveitis with no prior symptoms except for an itchy rash in the pelvic area. The patient was treated with IV penicillin after which full vision was restored. By presenting this case, we hope to stress the importance of adding syphilis in the differential diagnosis of men who have sex with men (MSM) who present with otherwise unexplained rash and/or uveitis.

## Introduction

Syphilis, a typically sexually transmitted disease caused by the spirochete bacterium Treponema pallidum, affects various organs resulting in a wide variety of presentations, hence the nickname "The Great Imitator". The broad symptomatology of the disease makes diagnosis challenging and sometimes delays treatment [1,2]. In recent years, the incidence of syphilis and other nationally notifiable diseases has increased worldwide and remains an important epidemiological problem [1-3].

Syphilis has four distinct clinical stages in the course of infection. The first stage, primary syphilis, traditionally manifests as one, round, painless genital or anal ulcer with a clean base called a “chancre” with localized painless adenopathy. Symptoms emerge about three weeks after inoculation (range 10 to 90 days). However, the classical chancre is often not recognized as a sign of syphilis as it spontaneously disappears after two to eight weeks. If left untreated, in about six to eight weeks the infection progresses to the secondary stage marked by a plethora of symptoms such as fever, fatigue, headache, weight loss, sore throat, patchy hair loss, lymphadenopathy, and a maculopapular rash on the flank, shoulders, arm, chest or back that often involves the soles of the feet and palms of the hands [4,5]. Spontaneous remission of symptoms marks the start of the latent stage. This asymptomatic and non-contagious stage has been documented to last for up to 10-30 years in a number of patients [6,7]. At this stage, the infection can relapse into the secondary stage or progress to tertiary syphilis. In this possibly fatal stage, the spirochetes can invade various organ systems such as the brain, nerves, eyes, heart, liver, bones, and joints [4,8].

The incidence of ocular syphilis is not clear. However, studies have reported a prevalence of ocular symptoms ranging from 0% to 7.9% of patients with secondary syphilis and up to 51% of neurosyphilis patients. Furthermore, the prevalence of ocular syphilis is higher in the men who have sex with men (MSM) population [3].

## Case presentation

**Figure 1:**
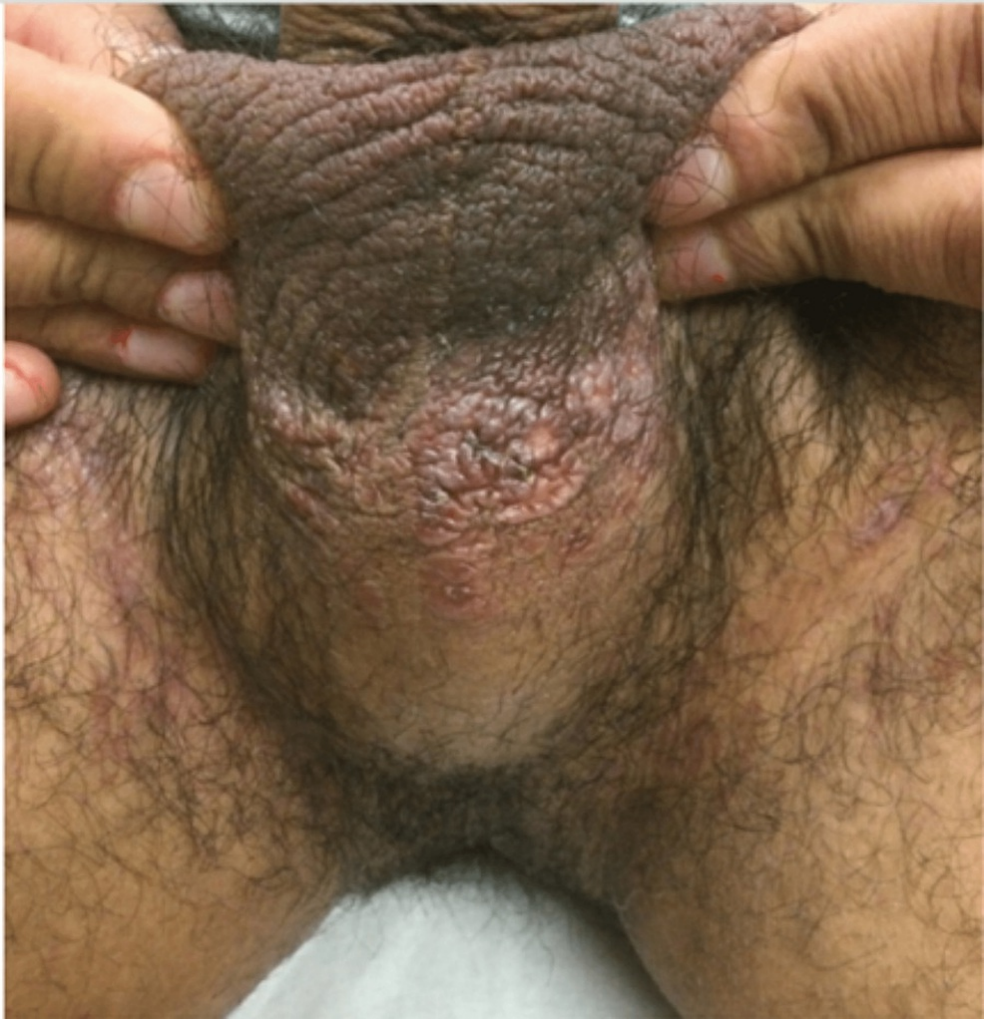
Rash involving the perineal area and scrotal skin

On August 19th, 2021, a 39-year-old male presented to the medical clinic for a pruritic rash involving the perineal area and scrotal skin that started several months ago (Figure 1). He denied any other symptoms and new exposures (soaps, lotions, detergents, foods, medication, insects, or animals). He had been treating the rash at home with ketoconazole cream and nystatin powder which temporarily improved the rash but got worse after. Lesions appeared as red patchy areas with certain areas with a dry texture. He also reported that he was an MSM with multiple sexual partners, although he was monogamous at the time of presentation, and inconsistent condom use. However, his chlamydia and gonorrhea nucleic acid amplification probe, hepatitis C antibody, HIV antibody/antigen combo screen, and syphilis T. pallidum total antibody with reflex screens from June 2021 were all negative. His vitals were within normal limits except for his BMI (Table 1) and no laboratory studies were ordered. He was prescribed two fluconazole (Diflucan) 150 mg tablets (one taken immediately and the other after 72 hours) and Ciclopirox (Loprox) 0.77% cream (applied topically twice daily) for suspected tinea cruris and referred to a dermatologist for further evaluation. A few weeks later, prior to the dermatologist visit, he reported that the rash returned after finishing the previous regiment ant treatment regimen was changed to ketoconazole (Nizoral) 2% cream and hydrocortisone 2.5% cream (applied topically three times a day for 60 days). He was ultimately diagnosed with lichen sclerosus by dermatology and was prescribed tacrolimus (Protopic) 0.1% ointment (apply to affected area one to two times daily). Six months into the start of symptoms his rash never fully recovered and developed into dermatitis. Subsequently, a trial of antibiotics (sulfamethoxazole-trimethoprim (Bactrim DS) 800-160 mg tablet, one tablet by mouth twice daily for five days) was done at this point which also did not improve the rash.

**Table 1:** Vital signs on initial encounter

Vital signs parameter	Finding
Blood pressure (Location: Right arm, Patient Position: Sitting)	122/80 mmHg
Pulse	76 beats per minute
Temperature	36 °C (96.8 °F)
Respiration	17 breaths per minute
Oxygen saturation	98%
Weight	95.6 kg (210 lb 12.2 oz)
Height	1.727 m (5' 7.99")
BMI	32.05 kg/m²

He was advised to get a repeat syphilis screen by the dermatologist, but it was not completed. Eight months after the start of symptoms he started experiencing blurriness, irritation, itchiness, and sensation of foreign bodies in both of his eyes with no other systemic symptoms. Subsequently he was referred to ophthalmology where he was diagnosed with bilateral posterior uveitis possibly secondary to a systemic condition (Table 2). He was sent to the emergency department for further management and care. Rapid plasma reagin (RPR) and syphilis antibody were ordered both of which came back positive. He was then admitted to the hospital for neurosyphilis/tertiary syphilis/ocular syphilis. After workups and labs (Table 3) he was started on penicillin 3 million units every four hours and later discharged on continuous IV penicillin G 18 million units in 0.9% NaCl IV through a peripherally inserted central catheter (PICC) line that he changed himself.

**Table 2:** Ophthalmological exam finding upon diagnosis of posterior uveitis CDR: cup-to-disc ratio

	Right eye	Left eye
Nerve	Sharp, pink, flat disc. Normal nerve fiber layer. CDR 0.3. Disc hyperemia	Sharp, pink, flat disc. Normal nerve fiber layer. CDR 0.3. Disc hyperemia
Vitreous	+1 Vitritis	+1 Vitritis
Retinal vessels	Normal caliber	Normal caliber
Macula	Flat	Flat
Periphery	Attached. Choroidal lesions scattered throughout posterior pole	Attached. Choroidal lesions scattered throughout posterior pole

**Table 3:** Workup and labs upon admission to hospital CMP: comprehensive metabolic panel, CBC: complete blood count, TSH: thyroid stimulating hormone, CRP: C-reactive protein, CSF: cerebrospinal fluid, WBC: white blood cells, RBC: red blood cells, VDRL: venereal disease research laboratory test,  LV EF: left ventricular ejection fraction

Lab/workup	Finding
CMP, CBC, TSH, CRP, procalcitonin	unremarkable
Lumbar Puncture/CSF	2.5 mL volume, colorless, clear, 17 WBC/mm3 (High; 75% lymphocytes, 25% monocytes), 14 RBC/mm3, glucose 46 mg/dL, protein 33 mg/dL, VDRL negative
CSF cultures/gram stain	No growth, no organisms seen on gram stain
CT head w/o contrast	Normal
Urinalysis/ urine culture	Normal/no growth
Blood culture	no growth at 5 days
Echocardiography	LV EF 57%, no significant valvular heart disease, and no clear vegetation seen
Chest x-ray	Normal

On the follow-up ophthalmology visit on June 6th, 2022, he reported improvement of visual problems and examination showed regression of posterior uveitis (Table 4) and a follow-up rapid plasma reagin test confirmed eradication of syphilis. 

**Table 4:** Ophthalmological exam finding on follow-up visit CDR: cup-to-disc ratio

	Right eye	Left eye
Nerve	Sharp, pink, flat disc. Normal nerve fiber layer. CDR 0.3. Regressing disc hyperemia.	Sharp, pink, flat disc. Normal nerve fiber layer. CDR 0.3. Regressing disc hyperemia.
Vitreous	+1 Vitritis	+1 Vitritis
Retinal vessels	Normal caliber	Normal caliber
Macula	Flat	Flat
Periphery	Attached. Regressing choroidal lesions scattered throughout posterior pole.	Attached. Regressing choroidal lesions scattered throughout posterior pole.

## Discussion

During any stage of syphilis, the central nervous system (CNS) may become involved [5]. Data from the Centers for Disease Control and Prevention (CDC) surveillance demonstrate that most suspected ocular syphilis cases occurred in the early phases (primary, secondary and early latent) [9]. The incidence of ocular syphilis is not well known; however, surveillance data have demonstrated that approximately 4.8% of individuals with syphilis had visual symptoms and 2.7% had objective findings consistent with ocular syphilis [3]. Previous reports have also demonstrated that approximately 5% of patients with secondary syphilis have early neurosyphilis indications, and ocular disease [6]. Furthermore, these reviews have revealed that the majority of ocular syphilis patients are MSM individuals and a recent skin rash was the most common reported symptom [3,9,10]. However, it should be noted that many patients may present with ocular symptoms of syphilis, especially HIV-infected patients with a CD4 count <350 cells/μL. Additionally, a prior report has shown that the most common diagnosis in the eye examination is uveitis (anterior uveitis, posterior uveitis, or pan uveitis) but retinitis and retinal detachment have also been reported in severe cases [3,11].

The coexistence of syphilis and HIV has long been demonstrated [4]. Previous reports have shown that approximately 50% of ocular syphilis patients were HIV positive [10]. In a study from North Carolina, 56% of patients with ocular syphilis were HIV infected, 31% of whom were simultaneously diagnosed with HIV and ocular syphilis [3]. Even though the rapid progression of syphilis in HIV-positive individuals has been documented, it is not clearly known to what extent HIV infection affects the onset or severity of ocular syphilis [7].

Prompt treatment of ocular syphilis is critical for visual function improvement. The CDC’s recommended treatment for adults with neurosyphilis including ocular syphilis is high-dose intravenous aqueous crystalline, or intramuscular procaine penicillin plus probenecid, for 10 to 14 days [5]. A report from the pre-antibiotic era has shown that without proper treatment, syphilitic iritis progresses to blindness in approximately 9%-12% of patients [12]. A retrospective review reported improved visual acuity after treatment with IV antibiotics while worse posttreatment visual acuity was noted when treatment was delayed >12 weeks after symptom onset [13].

## Conclusions

Known as “the great imitator”, syphilis presents itself in many forms and is notorious for posing diagnostic difficulties. The initial manifestations of the disease can be vague and varied. However, since syphilis is a treatable disease, we would like to emphasize the importance of having a high suspicion of syphilis when patients with multiple risk factors present with an atypical rash or uveitis, even though the initial testing might be negative. Clinicians must maintain a high index of suspicion and consider repeat testing, particularly in high-risk populations, as the outcome of untreated patients can be detrimental.
